# Design and Characterization of Double Layered Mucoadhesive System Containing Bisphosphonate Derivative

**DOI:** 10.1155/2013/604690

**Published:** 2013-12-19

**Authors:** Dhrubojyoti Mukherjee, Srinivasan Bharath

**Affiliations:** Department of Pharmaceutics, M. S. Ramaiah College of Pharmacy, Bangalore, Karnataka 560 054, India

## Abstract

The objective of this study is to evaluate the effect of formulation variables on different evaluation properties such as cumulative percentage release and swelling index in development of two layered buccal mucoadhesive system consisting of a highly water soluble drug risedronate sodium. The mucoadhesive systems were developed with varied concentrations of the polymers (1-2%) using plasticizer/permeation enhancer (25–50% w/w of polymer). Two layered films comprised of risedronate sodium with chitosan (85% deacetylated) and hydroxypropylmethyl cellulose (HPMC 4KM) interpolymer complex of different ratios were prepared by solvent casting method. An impermeable backing membrane of ethyl cellulose was incorporated into the films. The study shows the effect of multipolymeric films on the release of a bisphosphonates derivative. The optimized formulations showed films with uniform drug content (90.91 ± 0.17–105.53% ± 2.15), thickness (0.22 ± 0.01 mm to 0.31 ± 0.06 mm), mucoadhesivity (26 ± 3.61–42.33 ± 2.82 g), and controlled drug release profile up to a period of 10 hours. The films were also studied for swelling index, moisture uptake, viscosity, folding endurance, water vapor transmission rate, and mucoadhesive time.

## 1. Introduction

The selection of suitable polymers for manufacturing a drug delivery system is a major and important factor when formulation of controlled release buccal delivery systems for enhancing mucoadhesivity and obtaining controlled release profile is considered. A drug delivery system using a single polymer may not give the desired drug release profile when compared to blending polymers to get suitable and desired results with mucoadhesive drug delivery systems [1].

Buccal mucosa is an attractive route for the delivery of drugs through systemic route, because of its relatively good permeability with a rich blood supply. A drug can be easily applied and localized to the application site and can be removed from the site whenever necessary. Buccal films are highly flexible and easily tolerated by the patients. It also ensures accurate dosing of the drug. During the last decade, bioadhesive polymers received considerable attention as platforms for buccal controlled drug delivery due to their ability to localize the dosage form in specific regions to enhance bioavailability [2]. Due to their small size and thickness, they have improved patient compliance, compared to tablets. Since mucoadhesion implies attachment to the buccal mucosa, films can be formulated to exhibit a systemic or local action. Films releasing drug towards the buccal mucosa exhibit the advantage of avoiding the first pass effect by directing absorption through the venous system that drains from the cheek [3].

Drug delivery through buccal route provides direct access to the systemic circulation through the internal jugular vein, which bypasses the first pass metabolism leading to high bioavailability. Other advantages such as excellent accessibility, low enzymatic activity, suitability for drugs or excipients that mildly and reversibly damage or irritate the mucosa, painless administration, easy drug withdrawal, facility to include permeation enhancer in the formulation, and versatility in designing as multidirectional and unidirectional release systems for local and systemic actions make buccal drug delivery system as promising option for continued research [4].

Bioadhesive delivery systems have received considerable attention as promoters of absorption due to their ability to adhere to the mucin/epithelial cell surface and thereby anchor a dosage form at the site for optimum drug absorption and lead to an overall increase in bioavailability. Mucoadhesion utilizes the property of bioadhesion of certain water soluble or swellable polymers which become adhesive on hydration and hence can be used for targeting a drug to particular regions of the body where mucus or receptive epithelial cells are present for example, nasal, buccal, GIT, cervical, and vaginal. The formulation can remain attached for extended period of time and this may reduce toxic side effects and increase the therapeutic efficacy of the incorporated drug. Buccoadhesive delivery systems make use of polymers that are highly bioadhesive and do not dissolve before releasing the incorporated drug. Chitosan is gaining increasing importance in the pharmaceutical field due to its good biocompatibility and its nontoxicity and biodegradable property. Ethyl cellulose is a water insoluble polymer used as backing membrane for its film formability property and minimal toxicity [5].

For development of mucoadhesive, bilayered buccal films, chitosan, and HPMC-4KM inter-polymer complex was used. Because of the properties such as hydrophobicity, low water permeability, drug impermeability, and moderate flexibility ethyl cellulose was used as a backing membrane.

Osteoporosis and Paget's disease of bone are major problems in women and geriatric patients where antiresorptive agents are normally recommended. Bisphosphonates have an established role in the treatment of osteoporosis, Paget's disease of bone, malignant hypocalcaemia during myeloma, osteolytic bone metastasis, and fibrous dysplasia of bone. Despite their benefits, bisphosphonates suffer from very poor oral bioavailability (<1%). Higher localized concentration of bisphosphonates has resulted in severe gastrointestinal side effects such as dysphagia, esophagitis, and gastric ulceration [6].

The objective of the present work was to develop two layered mucoadhesive formulations with varied concentration of a blend of hydrophilic and hydrophobic polymer (1-2%) using plasticizer/permeation enhancer (25–50% w/w of polymer) and Also to evaluate the prepared formulations for various physicochemical, drug release, and compatibility characteristics.

## 2. Materials and Methods

Risedronate sodium was a gift sample from Fleming Laboratories, Hyderabad, India. Chitosan was procured from the Indian Institute of Fisheries, Cochin, India. HPMC-4KM and Ethyl cellulose were obtained commercially from SD Fine Chemicals, India. All other reagents and chemicals used were of analytical reagent grade.

### 2.1. Preparation of Mucoadhesive Bilayered Buccal Films [2, 4]

*Backing Layer*. For the preparation of the formulations, glass Petri plates of 9 cm diameter were used as a casting surface. A solution containing 1 g of ethyl cellulose, with diethyl phthalate 2% w/w of the polymer as plasticizer in 20 mL of acetone, was poured slowly to the glass Petri plate and air dried overnight.

*Mucoadhesive Layer Containing Drug*. The drug risedronate sodium was dissolved in a solution of HPMC-4KM in a specified quantity of purified water. Chitosan was separately dissolved in specified volume of 1% v/v acetic acid under constant stirring till clear solution was obtained. The drug-polymer solution and chitosan solution were uniformly mixed together in a magnetic stirrer and propylene glycol was added to the solution. The resultant solution was then casted on the preformed backing layer of ethyl cellulose and allowed to dry by placing an inverted funnel onto the petri plate undisturbed at room temperature. The dried films were stored in desiccator until further used.

The compositions of two layered buccal films are as given in Table 1.

### 2.2. Evaluation Studies

#### 2.2.1. Drug Polymer Compatibility Study

*The Drug*. polymer interaction study was carried out by analyzing the pure drug, polymer, and the drug: polymer physical mixture (1 : 1) using a KBr pellet and scanned from 400 to 4000 cm^−1^ using FTIR (Shimadzu S-1601, Japan).

#### 2.2.2. Mass Uniformity and Thickness Determination [4, 7]

Five randomly selected films were taken from each formulation and weighed and the mean was calculated. Film thickness was determined by a screw gauge and recorded as the mean of five measurements representing the four corners and the center of each film.

#### 2.2.3. Swelling Index Determination [8, 9]

The films were weighed, placed in a 2% agar gel plate, and incubated at 37 ± 1°C. At regular time intervals, the films were removed from the plates and excess surface water was removed carefully using a filter paper. The swollen films were then reweighed and the degree of swelling was calculated using the following formula:

(1) $$\begin{array}{c} \text{Degree}\;\text{of}\;\text{Swelling}=\frac{\text{Wet}\;\text{weight}-\text{Initial weight}}{\text{Initial}\;\text{weight}}. \end{array}$$

#### 2.2.4. Folding Endurance Test [8]

The number of times the film could be folded at the same place till it broke gave the value of the folding endurance. The test was performed by repeatedly folding one film at the same place till it broke or folded up to 300 times at the same place without breaking gave the value of the folding endurance of the film.

#### 2.2.5. Drug Content Analysis [8, 10]

For determination of content uniformity of the films five films were taken. Drug was extracted from the films by sonicating and dissolving in 50 mL of phosphate buffer (pH 7.4) and filtering through whatmann filter paper (0.45 *μ*m). The resultant filtrate was diluted to 250 mL and absorbance was recorded at 262 nm using UV-Spectrophotometer (Shimadzu UV 1700, Japan).

#### 2.2.6. *Ex Vivo* Mucoadhesive Strength Test [11, 12]

**Figure 1:**
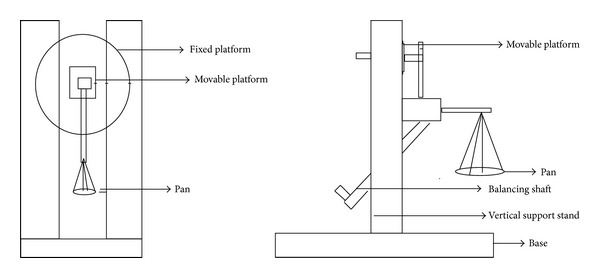
Mucoadhesive strength test apparatus.

The mucoadhesive strength of the films were measured using a design and fabricated mucoadhesive strength test apparatus (Figure 1). It consists of a metal base holding a vertical support stand. Onto the stand there are two platforms; one is fixed and another is movable. The movable platform is in turn balanced onto the equipment with a balancing shaft. A pan is present in the apparatus to put the weight and measure the bioadhesive strength. Porcine buccal mucosa was obtained from a local slaughter house and used within 2 hours of sacrificing the animal. The underlying fat and loose tissues were separated from the mucosal membrane and washed with distilled water and then with pH 6.8 phosphate buffer at 37°C.

After the preparation of the tissue, a mucosal layer of 3 cm^2^ was fixed to the immovable platform of the apparatus with cyanoacrylate gum. In a similar way the sample film was glued to the movable platform. For initial hydration and swelling, the exposed film surface was moistened with 15 *μ*L of phosphate buffer and left for 30 s. The movable platform then slowly moved towards the fixed platform in a horizontal direction and was brought in contact with the mucosal surface. A preload of 20 g was placed over the movable platform for 3 minutes as the initial pressure for proper attachment of the film with the mucosal membrane. Proportionately at a definite interval of time, weights were added onto the pan attached with the movable platform. The total weight required for complete detachment of the film was recorded and the different mucoadhesive strength characteristics were calculated as follows:

(2) $$\begin{array}{c} \text{Force}\;\text{of}\;\text{adhesion}=\frac{\text{Bioadhesive}\;\text{strength}\times 9.81}{1000},\\ \text{Bond}\;\text{strength}=\frac{\text{Force}\;\text{of}\;\text{adhesion}}{\text{Film}\;\text{surface}\;\text{area}}, \end{array}$$

where mucoadhesive strength is the mass in grams required to detach the film from the mucosal surface. Bond strength is the representation of the area under the work or energy required for detachment of the two systems (mucin/polymeric film). The results of mucoadhesive strength are given in Table 2.

#### 2.2.7. *Ex Vivo* Mucoadhesion Time [12]

The study was performed by application of the films onto freshly cut porcine buccal mucosa. The fresh buccal mucosa was fixed in the inner side of the beaker, above 2.5 cm from the bottom with cyanoacrylate glue. The drug layer side of each film was wetted with one drop of isotonic phosphate buffer pH 6.8 and pasted to the porcine buccal mucosa by applying a light force with a fingertip for 30 seconds. The beaker was filled with 200 mL of phosphate buffer pH 6.8 and was kept at 37 ± 1°C. After 2 minutes, a 50 rpm stirring rate was applied to simulate the buccal cavity environment, and film adhesion was monitored up to 12 h. The time required for the film to detach from the buccal mucosa was recorded as the mucoadhesion time.

#### 2.2.8. *Ex Vivo* Permeation Studies [2, 4]

The permeation study was carried out through porcine buccal mucosa, using a Keshary Chien glass diffusion cell. The mucosa was mounted between the donor and receptor compartment. The formulation with drug layer was placed on the mucosa and the compartments were clamped together. The donor compartment was filled with 1 mL of phosphate buffer pH 6.8. The receptor compartment (15 mL capacity) was filled with phosphate buffer pH 7.4 maintained at 37 ± 0.2°C and the hydrodynamics in the receptor compartment was maintained by stirring with a magnetic bead at 50 rpm. One mL sample was withdrawn at predetermined time interval and analyzed for drug content at 262 nm.

#### 2.2.9. Water Vapour Transmission Rate [13]

Glass vials of equal diameter were used as transmission cells. About 1 g of anhydrous calcium chloride was taken in the cells and the polymer films were fixed onto the brim of the transmission cells. Each transmission cell containing the polymer film was weighed and kept in a closed desiccator containing saturated solution of potassium chloride to maintain the humidity. The cells were taken out and reweighed after an interval of 24 hours up to 7 days of storage. The amount of water vapour transmitted was calculated using the following formula:

(3) $$\begin{array}{c} \text{Water}\;\text{vapour}\;\text{transmission}=\frac{WL}{S}, \end{array}$$

where *W* is the vapour transmission rate, expressed as the grams of moisture, *L* is the thickness of the film in cm, and *S* is the exposed surface area in cm^2^.

#### 2.2.10. Percent Moisture Absorption Study [10, 13]

The weighed films were placed in a desiccator containing saturated solution of potassium chloride to maintain the humidity. After an interval of every 24 hours the weight of the films was checked until the films show a constant weight. The percentage moisture absorption was calculated by

(4) % Moisture absorption=(Final weight−Initial weight)Initial  weight×100.

#### 2.2.11. Surface pH Determination [14]

A combined glass electrode was used for this purpose. The films were allowed to swell by keeping them in contact with 1 mL of pH 6.8 phosphate buffer for 2 h at room temperature and pH was noted down by bringing the electrode in contact with the surface of the film, allowing it to equilibrate for one minute.

## 3. Results and Discussion

The present study was conducted based on a preliminary study performed on the bilayered mucoadhesive delivery system prepared with chitosan and HPMC-4KM interpolymer complex, where the polymer concentration limits were 0.33 g (lower) and 0.67 g (higher). In this work, 14 formulations were developed with different polymer and plasticizer concentration. Different concentrations of two polymers, chitosan and HPMC-4KM, at a concentration range of 1-2% w/v were prepared by solvent casting method and the different film evaluation parameters were studied. Propylene glycol was used as a plasticizer and the concentration was varied between 25 and 50% w/w of the total polymer concentration.

### 3.1. Drug-Polymer Compatibility Study

**Figure 2:**
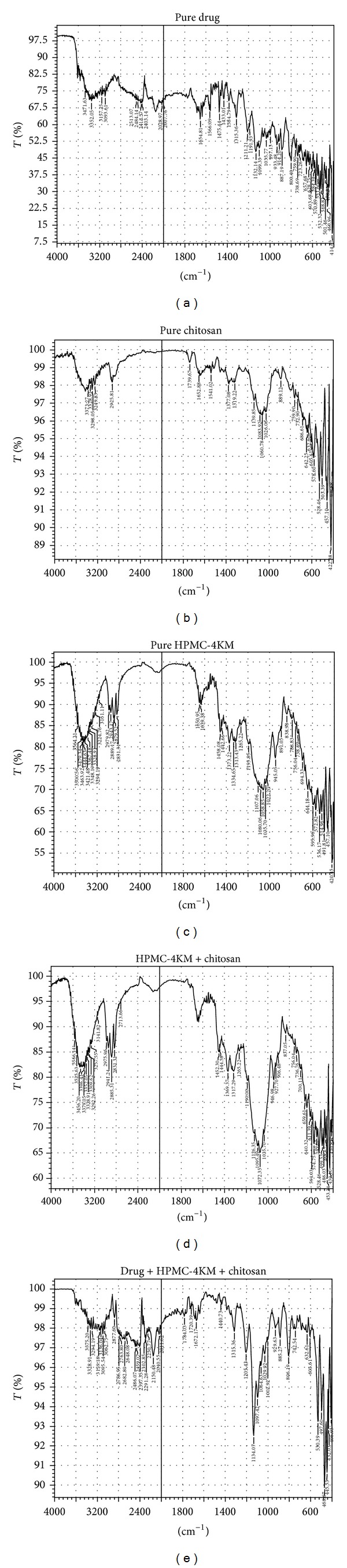
FTIR spectra of (a) pure drug, (b) pure chitosan, and (c) pure HPMC 4KM. (d) physical mixture (1 : 1) HPMC 4KM and chitosan and (e) physical mixture of drug : polymer (1 : 1) [Drug + HPMC4KM + chitosan].

FTIR studies were conducted for drug-polymer mixture compatibility study. The characteristic peaks of the pure drug, polymers and drug polymer mixture are shown in Figure 2 and in Table 5. The IR spectra of physical blend of the polymers and the drug with the polymers showed neither shift nor disappearance of characteristic peaks suggesting that there is no interaction between drug and polymers, and they are very much in conformity with the standard reference spectra.

### 3.2. Morphology, Mass, and Thickness Evaluation

The optimized formulations had good flexibility and transparency and smooth and uniform surface. The film mass and thickness ranged from 0.084 ± 0.014 g to 0.109 ± 0.008 g and from 0.22 ± 0.012 mm to 0.31 ± 0.006 mm, respectively, as shown in Table 2. The formulation with higher mass and thickness may be due to the presence of uneven surface of ethyl cellulose, which is used as a backing membrane. The mass and thickness range was found to be satisfactory considering the fact that the films are of size 1.5 cm × 1.5 cm, which should not cause any inconvenience to the patient after application.

### 3.3. Drug Content, Surface pH, and Folding Endurance

The drug content in the film showed optimum and uniform drug loading ranging from 90.91 ± 0.172% to 105.53 ± 2.155%. The surface pH ranges from 5.36 ± 0.530 to 7.19 ± 0.698. Considering the oral pH ranging from 5 to 7.5, the pH range of the buccal films should not cause any harmful effect to the patient and also will not cause any problem in the drug release. None of the formulations broke below 300 folding at the same place, suggesting that the films are not brittle and have higher flexibility. The results are shown in Table 2.

### 3.4. Percentage Moisture Absorption and Water Vapour Transmission

A direct correlation can be suggested between moisture absorption and vapour transmission with stability and drug release mechanism. Percentage moisture absorption ranges from 6.370 ± 0.120% to 18.050 ± 0.096% and water vapour transmission from 0.0016 ± 0.0002 g/cm^2^ to 0.106 ± 0.0002 g/cm^2^. Very high moisture content and moisture permeation may lead to deterioration of the film by microbial contamination. Low moisture content and moisture permeation may lead to the films being brittle. A subsequent moisture content and fluid imbibition is necessary for the films during drug release, where the fluid gets channelize inside and solubilise the drug in the polymer matrix and there by leading to release of the drug. Formulations with higher concentrations of HPMC-4KM and high plasticizer concentration (A4, A6, A11, A13, and A14) lead to higher moisture absorption and water vapour transmission. The values are shown in Table 2.

### 3.5. Bioadhesive Parameters of the Films

Mucoadhesion is a phenomenon occurs by adhesion between the polymer matrix in the formulations and the mucous layer. All the formulations showed good mucoadhesion behaviour. Films with higher concentration of chitosan showed higher *ex-vivo* mucoadhesive strength, force of adhesion, and bond strength. All the films were observed to have mucoadhesion time between 7.13 ± 1.75 and 12.32 ± 0.21 hours. Formulation A9 with 1% total polymer concentration and formulation A13 with 1.75% total polymer concentration are having highest mucoadhesive properties. Both chitosan and HPMC-4KM are known to have good mucoadhesive properties. A proper polymeric combination with a higher concentration of plasticizer (50% for both the formulations) may have attributed to a higher mucoadhesion. The higher mucoadhesive property may also be due to interpolymer complexation of chitosan and HPMC-4KM, both of which are known to have very good mucoadhesive properties. Mucoadhesive properties of the prepared films are given in Table 3.

### 3.6. Swelling Parameter of the Films

**Figure 3:**
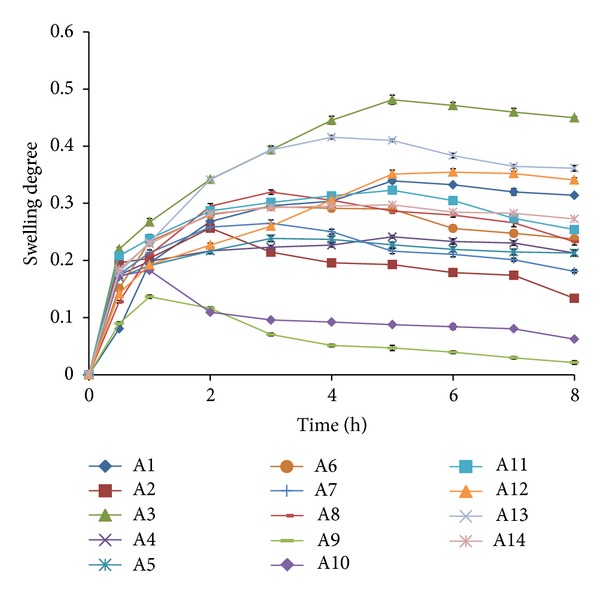
Swelling degree of the bilayered mucoadhesive films.

Swelling index was a criterion for the optimization. None of the films shows a very high swelling degree to cause discomfort to the patient. The formulations with a higher concentration of HPMC-4KM and plasticizer showed a higher swelling. This may be due to the fact that the polymer and the plasticizer are hydrophilic in nature and absorbs water, which leads to a higher swelling. When the total polymer concentration of the formulations is low in combination with a lower plasticizer concentration then the swelling degree was also low (A2, A9, and A10 with a total polymer concentration of 1.5%, 1%, and 1.5% resp.). The highest swelling was seen for formulations A3 and A13. Both these formulations contain a high ratio of water soluble polymer HPMC-4KM and a high concentration of plasticizer/permeation enhancer, which assist permeation of water into the films. Most of the films showed a significant amount of erosion after a maximum period of swelling. Formulations A2, A9, and A10 showed maximum swelling upto a period of 1 to 2 hours and then showed erosion up to 8 hours. The swelling degree of the films is represented in Figure 3.

### 3.7. *Ex-Vivo* Permeation Profile of the Films

**Figure 4:**
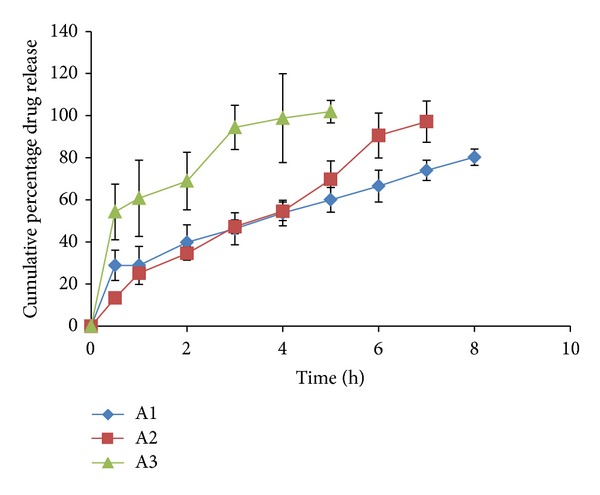
*Ex vivo* drug release profile of formulations A1, A2, and A3.

**Figure 5:**
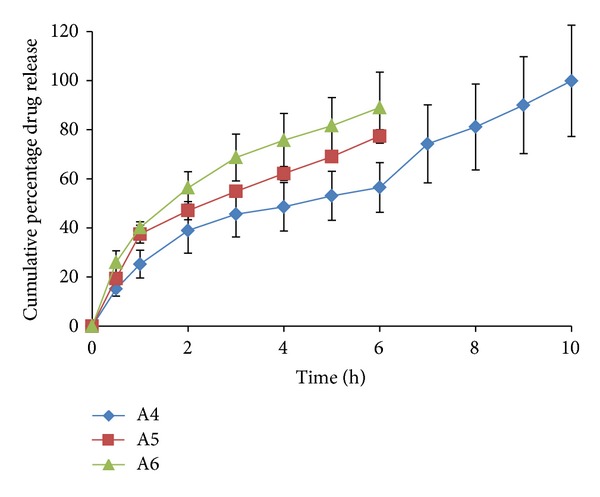
*Ex vivo* drug release profile of formulations A4, A5, and A6.

**Figure 6:**
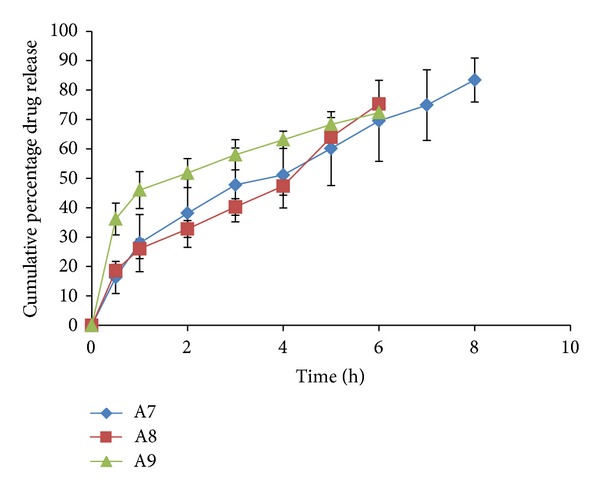
*Ex vivo* drug release profile of formulations A7, A8, and A9.

**Figure 7:**
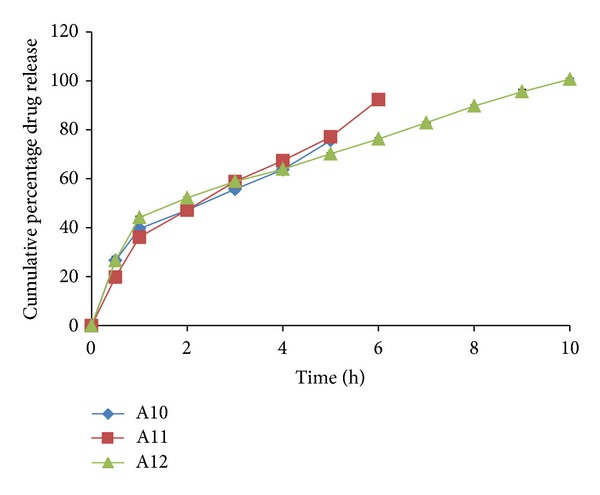
*Ex vivo* drug release profile of formulations A10, A11, and A12.

**Figure 8:**
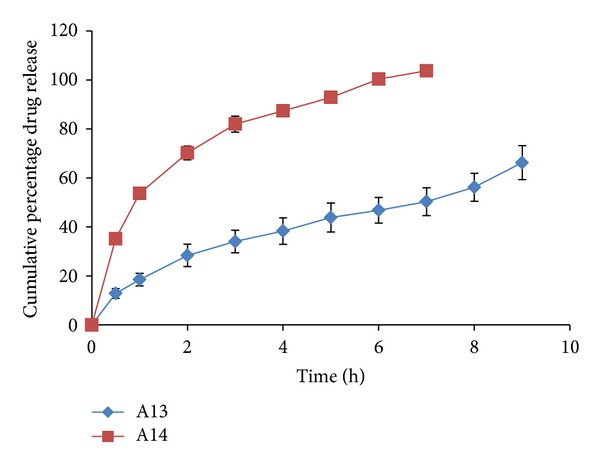
*Ex vivo* drug release profile of formulations A13 and A14.

All the formulations were observed to have a better release property than previously studied preliminary formulation trials. The cumulative % drug release ranged from 66.244 ± 6.9 to 103.72 ± 0.39% for all the prepared double layered film formulations. The drug release was observed to increase with an increasing concentration of polymer HPMC-4KM. This may be attributed to hydrophilic nature of the polymer which has an increased capacity to imbibe and absorb water, thereby promoting dissolution and release of highly water soluble drug risedronate sodium. The hydrophilic polymer also would dissolve, creating pores and channels which would allow an easy access to water inside the formulation and there by a higher drug release. It has been observed that with a total polymer concentration of 1.5% (formulations A2 and A3 show a drug release of 97.137 ± 9.815 up to a period of 8 hours and 101.883 ± 5.385 up to a period of 5 hours resp.) and with polymer concentration 1.75% (formulation A14 shows a drug release of 103.720 ± 0.399 up to a period of 7 hours) the formulations showed highest drug release. Formulation A12 with a total polymer concentration of 2% and plasticizer concentration of 25% showed a release of 100.613 ± 0.353 up to a period of 10 hour. The reason could be the high percentage of moisture absorption 10.92 ± 0.283 and comparatively high water vapour transmission 0.0039 ± 0.0038. Due to high water permeability and erosion property of chitosan (swelling index), there was an easy access for water and solubilisation of drug and release. All other formulations showed a higher release (more than 70%) when compared to the previously studied formulations. The percentage cumulative release profiles of the formulations are shown in Figures 4, 5, 6, 7, and 8.

### 3.8. Release Kinetics of the Two Layered Films

To analyse and understand the release mechanism of the drug from the films, the *ex-vivo* drug release data were computed using PCP DISSO V2 software. The release mechanism from the formulations can be interpreted from the release exponent (*n*) values. Formulations A1 and A9 show the value of *n* as 0.45 (*n* = 0.452) and 0.5 (*n* = 0.5), respectively, which suggests Fickian diffusion as the release kinetics. For the remaining formulations the release exponent values ranged from 0.53 to 0.679 suggesting anomalous (non-Fickian transport) diffusion mechanism. All the formulations showed a closed similarity and quality adjustment with Highuchi release model as indicated in Table 4. Formulation A3 showed a higher *R* ^2^ value in Highuchi (0.9978) than in Korsmeyer-Peppas (0.8965) release kinetics. This suggests that the water soluble drug is dispersed uniformly in the swellable polymer matrix.

## 4. Conclusion

It may be concluded that buccal route is one of the alternatives available for administration of risedronic acid, a bisphosphonate derivative. As per the characteristics features of the films observed in all the formulations, a proper combination of the polymers with permeation enhancer is necessary to achieve permeation of drug through buccal mucosa in a controlled manner. The formulations A2, A3, A12, and A14 containing a suitable proportion of the polymers with an optimum proportion of permeation enhancer showed good swelling and mucoadhesive property with 90−100% of drug release within a period of 8 to 12 hours.

## Figures and Tables

**Table 1:** Compositions of buccal films.

Batch code	Risedronate sodium (g)	Chitosan (%w/v)	HPMC-4KM (%w/v)	Ethyl cellulose (g)	Propylene glycol (%w/w)	Acetic acid 1% V/V (mL)	Purified water (mL)
A1	0.28	1	1	1	50	25	25
A2	0.28	1	0.5	1	25	30	20
A3	0.28	0.5	1	1	50	20	30
A4	0.28	0.75	0.88	1	37.5	20	30
A5	0.28	0.5	0.5	1	25	25	25
A6	0.28	0.75	0.75	1	25	25	25
A7	0.28	0.5	0.75	1	37.5	20	30
A8	0.28	1	0.5	1	50	30	20
A9	0.28	0.5	0.5	1	50	25	25
A10	0.28	0.5	01	1	25	20	30
A11	0.28	0.75	0.5	1	37.5	30	20
A12	0.28	1	1	1	25	25	25
A13	0.28	0.75	1	1	50	20	30
A14	0.28	1	0.75	1	37.5	30	20

**Table 2:** Evaluation parameters of mucoadhesive films.

Batch code	Thickness (mm) mean ± S.D	Mass uniformity (g) mean ± S.D	% drug content mean ±S.D	Surface pH mean ± S.D	Folding endurance mean ± S.D	%moisture absorption mean ± S.D	Vapour transmission mean ± S.D
A1	0.23 ± 0.017	0.108 ± 0.004	92.81 ± 0.378	7.03 ± 0.249	>300	9.424 ± 0.227	0.0033 ± 0.0003
A2	0.26 ± 0.017	0.092 ± 0.008	99.8 ± 0.390	5.78 ± 0.050	>300	9.976 ± 0.125	0.0033 ± 0.0002
A3	0.24 ± 0.020	0.084 ± 0.014	93.36 ± 2.915	6.09 ± 0.146	>300	10.620 ± 0.228	0.0018 ± 0.0004
A4	0.22 ± 0.012	0.093 ± 0.008	91.58 ± 0.671	6.72 ± 0.048	>300	18.050 ± 0.096	0.0033 ± 0.0003
A5	0.23 ± 0.012	0.086 ± 0.006	93.73 ± 6.091	6.87 ± 0.346	>300	6.870 ± 0.450	0.0028 ± 0.0001
A6	0.25 ± 0.015	0.094 ± 0.009	91.49 ± 1.904	6.35 ± 0.483	>300	8.330 ± 0.321	0.0065 ± 0.0028
A7	0.23 ± 0.012	0.092 ± 0.003	97.02 ± 5.693	7.19 ± 0.698	>300	6.370 ± 0.120	0.0057 ± 0.0002
A8	0.26 ± 0.020	0.102 ± 0.004	90.91 ± 0.172	5.93 ± 0.366	>300	9.860 ± 0.225	0.0026 ± 0.0001
A9	0.22 ± 0.021	0.107 ± 0.013	94.65 ± 5.369	6.05 ± 0.422	>300	7.370 ± 0.218	0.0022 ± 0.0031
A10	0.27 ± 0.035	0.099 ± 0.011	94.58 ± 5.468	6.70 ± 0.233	>300	7.630 ± 0.198	0.0046 ± 0.0003
A11	0.25 ± 0.010	0.091 ± 0.009	93.45 ± 2.060	5.88 ± 0.301	>300	8.710 ± 0.278	0.0053 ± 0.0021
A12	0.28 ± 0.015	0.108 ± 0.008	105.53 ± 2.155	7.01 ± 0.089	>300	10.920 ± 0.283	0.0039 ± 0.0038
A13	0.31 ± 0.006	0.109 ± 0.008	91.89 ± 0.828	6.93 ± 0.210	>300	10.370 ± 0.089	0.0106 ± 0.0002
A14	0.24 ± 0.061	0.093 ± 0.011	100.91 ± 1.884	5.36 ± 0.530	>300	11.480 ± 0.158	0.0016 ± 0.0002

**Table 3:** Bioadhesive parameters of the mucoadhesive films.

Batch code	*Ex vivo* mucoadhesive strength (g) mean ± S.D	Force of adhesion (N) mean ± S.D	Bond strength (Nm^−2^) mean ± S.D	Mucoadhesion time (h) mean ± S.D
A1	30.67 ± 12.89	0.301 ± 0.127	13.37 ± 5.62	8.29 ± 0.52
A2	26.33 ± 2.31	0.258 ± 0.023	11.48 ± 1.00	8.36 ± 0.56
A3	27.67 ± 2.08	0.271 ± 0.020	12.06 ± 0.91	7.50 ± 0.66
A4	35.67 ± 12.90	0.350 ± 0.127	15.55 ± 5.62	12.13 ± 0.84
A5	35.01 ± 3.23	0.343 ± 0.130	15.26 ± 5.76	7.33 ± 1.23
A6	29.33 ± 1.16	0.288 ± 0.011	12.78 ± 0.50	10.44 ± 0.29
A7	37.67 ± 4.79	0.370 ± 0.106	16.42 ± 4.70	9.22 ± 1.23
A8	31.67 ± 7.64	0.311 ± 0.076	13.80 ± 3.33	8.44 ± 0.9
A9	42.33 ± 6.62	0.415 ± 0.163	18.45 ± 7.24	7.13 ± 1.75
A10	34.13 ± 4.15	0.334 ± 0.100	14.823 ± 4.42	7.39 ± 1.26
A11	39.26 ± 5.53	0.350 ± 0.141	15.55 ± 6.26	11.50 ± 0.98
A12	28.33 ± 1.53	0.278 ± 0.015	12.35 ± 0.66	10.44 ± 0.46
A13	46.67 ± 2.82	0.458 ± 0.204	20.347 ± 9.07	12.32 ± 0.21
A14	26.94 ± 3.61	0.255 ± 0.035	11.33 ± 1.57	8.11 ± 0.89

**Table 4:** Results of curve fitting analysis.

Batch code	Korsmeyer-Peppas K (h^−*n*^)	*R* ^2^	Matrix model K (%, h^−1/2^)	*R* ^2^	Release exponent (*n*)
A1	29.51 ± 0.45	0.9972	27.64 ± 0.13	0.9979	0.452
A2	23.73 ± 0.86	0.9927	31.80 ± 0.30	0.9631	0.679
A3	26.65 ± 1.21	0.8965	52.51 ± 0.22	0.9978	0.540
A4	23.55 ± 0.33	0.9924	27.64 ± 0.10	0.9783	0.580
A5	29.26 ± 0.76	0.9844	31.56 ± 0.33	0.9938	0.573
A6	36.64 ± 0.58	0.9941	37.55 ± 0.05	0.9966	0.530
A7	24.59 ± 0.66	0.9964	27.81 ± 0.21	0.9949	0.579
A8	24.12 ± 0.53	0.9868	26.77 ± 0.09	0.9766	0.567
A9	32.33 ± 1.22	0.9905	32.83 ± 0.96	0.9327	0.501
A10	36.92 ± 0.59	0.9944	33.56 ± 0.11	0.9902	0.589
A11	32.26 ± 0.88	0.9950	34.91 ± 0.12	0.9940	0.559
A12	38.28 ± 0.78	0.9931	32.26 ± 0.58	0.9882	0.612
A13	18.06 ± 0.62	0.9973	19.90 ± 0.25	0.9932	0.557
A14	30.74 ± 0.55	0.9938	44.26 ± 0.55	0.9808	0.597

**Table 5:** IR spectral studies.

Sample	Wave number (cm^−1^)	Observation
Chitosan	2840–3000	C–H stretch
	1830–1870	C=O
	3350–3310	N–H stretch
	3420–3590	O–H stretch
	1020–1275	C–O–C

HPMC-4 KM	3570–3200	O–H stretching
	2980–2950	Aliphatic C–H stretching

Risedronate sodium	3080–3010	Aromatic C-H stretch
	1600–1430	C=C and C=N stretch
	3610–3330	O-H stretch
	~1150	Aliphatic P=O Stretch
	~1190	Aromatic P=O stretch
